# Prevalence and genotype distribution of high-risk HPV in Sarawak, East Malaysia

**DOI:** 10.1186/s12879-026-14020-z

**Published:** 2026-07-17

**Authors:** Cassandra Sheau Mei Chee, Shirley Siang Ning Tan, Mohamad Adam Bujang, Izzati Binti Wan Maharuddin, Adam Malik Bin Ismail, Rehn Peterson Peter, Rahmah Binti Rashid, Nur Fatihah Oh Abdullah, Mohammad Nazarudin Bin Bahari, Veronica Lugah, Keh Li Yew, Pei Jye Voon, Melissa Siaw Han Lim

**Affiliations:** 1 Faculty of Medicine and Health Sciences, Universiti Malaysia Sarawak, Kota Samarahan, Malaysia; 2 Clinical Research Centre, Institute for Clinical Research, National Institutes of Health, Sarawak General Hospital, Ministry of Health, Kuching, Malaysia; 3 Department of Pharmacy, Sarawak General Hospital, Ministry of Health, Kuching, Malaysia; 4 Department of Radiotherapy, Oncology and Palliative Care, Sarawak General Hospital, Ministry of Health, Kuching, Malaysia; 5 Department of Pathology, Sarawak General Hospital, Ministry of Health, Kuching, Malaysia; 6 Family Health Development Division, Department of Public Health, Sarawak State Health Department, Ministry of Health, Kuching, Malaysia

**Keywords:** Human papillomavirus (HPV), High-risk HPV, HPV genotype, Cervical cancer screening, Community-based screening, Sarawak, Malaysia, Epidemiology

## Abstract

**Background:**

Cervical cancer remains a significant public health burden in Malaysia, with persistent infection by high-risk papillomavirus (hrHPV) as the primary cause. However, community-based data on HPV prevalence and genotype distribution, particularly in East Malaysia, remain limited. This study aimed to determine hrHPV prevalence, genotype distribution, and associated factors in a large community-based screening cohort in Sarawak.

**Methods:**

A retrospective cross-sectional study was conducted between December 2025 and February 2026, using data from women who participated in community-based cervical screening programs across Sarawak. Cervicovaginal samples were collected via cervical swab, liquid-based preparation, or conventional pap smear, and analyzed using the Anyplex™ II HPV HR Detection assay. HrHPV prevalence, genotype distribution, and associations with demographic and clinical factors were analysed using descriptive statistics and logistic regression.

**Results:**

A total of 7,781 participants were included, with an overall hrHPV prevalence of 9.2%. The most frequently detected genotypes were HPV52, followed by HPV68 and HPV56, while HPV16 and HPV18 were less prevalent. Geographic variation was observed across divisions, with prevalence ranging from 3.6% to 11.7%. Ethnic variation was also noted, although differences among major ethnic groups were modest. Of these 286 participants, 82.2% had negative for intraepithelial lesion or malignancy (NILM), while 17.8% had abnormal Pap smear findings.

**Conclusion:**

This study provides important community-based evidence on hrHPV epidemiology in Sarawak, highlighting regional and genotype-specific variation. The predominance of non-16/18 genotypes underscores the importance of continued surveillance to inform vaccination and screening strategies in Malaysia.

**Supplementary Information:**

The online version contains supplementary material available at https://doi.org/10.1186/s12879-026-14020-z.

## Introduction

Cervical cancer remains a significant global health burden, particularly in low- and middle-income countries, with persistent high-risk human papillomavirus (hrHPV) infection being the primary etiological factor [1]. Despite the availability of effective screening programs, such as the pap smear, national coverage rates often remain suboptimal, particularly in rural and geographically isolated communities [2]. This disparity in screening uptake highlights the critical need for comprehensive epidemiological studies to understand HPV prevalence and genotype distribution in underserved populations [3]. Such investigations are crucial for informing the development of targeted, region-specific screening strategies and vaccination programs aimed at reducing cervical cancer incidence [4, 5].

In Malaysia, cervical cancer continues to contribute substantially to morbidity and mortality among women, ranking as the third leading cause of cancer-related deaths [6]. Several studies have reported HPV prevalence and genotype distribution among specific cohorts within Malaysia, however, these data are largely derived from hospital-based cohorts with limited geographic representation [2]. As a results, there is a substantial gap in understanding the broader community-level HPV epidemiology, particularly in ethnically diverse and geographically challenging regions like Sarawak, East Malaysia [3]. Our recent systematic review of HPV prevalence in Malaysia highlighted substantial geographical disparities in available data, with the majority of studies originating from Peninsular Malaysia and limited representation from East Malaysia, particularly Sarawak [3].

Sarawak, the largest state in Malaysia, recorded among the highest cervical cancer incidence rates in the country, with an incidence of 12.1 per 100,000 women reported in recent estimates [7]. The state presents unique public health challenges due to its vast geographical landscape, dispersed rural communities, and limited accessibility to healthcare services in certain interior regions [7]. Many populations reside in remote areas that require extensive travel by river or road to access healthcare facilities, potentially contributing to lower participation in routine cervical cancer screening programmes and delayed follow-up care [8]. These logistical barriers may disproportionately affect women from underserved communities and contribute to inequities in cervical cancer prevention efforts across the state.

In addition to geographical challenges, Sarawak is characterised by extensive ethnic diversity, comprising numerous indigenous and non-indigenous populations with distinct sociocultural backgrounds. Differences in health-seeking behaviour, socioeconomic status, cultural perceptions toward screening, and healthcare accessibility may influence HPV transmission dynamics and screening uptake across populations [3]. The influence of these demographic factors on the prevalence of different HPV genotypes further underscores the importance of region-specific epidemiological studies [3, 9]. However, despite these unique demographic and geographical characteristics, comprehensive population-based studies on HPV prevalence and genotype distribution in Sarawak remain limited.

Several prophylactic HPV vaccines are currently available, including the bivalent vaccine targeting HPV16 and HPV18, the quadrivalent vaccine targeting HPV6, HPV11, HPV16, and HPV18 and the nonavalent vaccine, which additionally provides protection against HPV31, HPV33, HPV45, HPV52, and HPV58 [10]. These vaccines have demonstrated substantial effectiveness in reducing infections caused by vaccine-targeted HPV genotypes and associated cervical precancerous lesions. However HPV genotype distribution varies across populations, highlighting the importance of continued surveillance to monitor circulating HPV types and inform vaccination strategies [11]. Malaysia introduced the national HPV vaccination programme for adolescent girls in 2010, resulting in improved vaccine coverage nationwide [7, 12]. Nevertheless, coverage of cervical cancer screening remains suboptimal, highlighting the importance of continued HPV surveillance and effective screening strategies to support cervical cancer elimination efforts [12]. Disparities in vaccine uptake and access to cervical screening services may still exist between urban and rural populations, particularly in geographically challenging regions such as Sarawak [7]. Moreover, regional variation in HPV genotype distribution may influence the long-term effectiveness of vaccination strategies, especially in populations where non-16/18 hrHPV genotypes are prevalent [13, 14]. Continuous surveillance of HPV prevalence and genotype distribution is therefore essential to support evidence-based cervical cancer prevention policies and to monitor the impact of vaccination programmes over time.

Therefore, this study aimed to determine the prevalence of HPV infection and describe the genotype in a large community-based screening cohort in Sarawak. Additionally, we evaluated demographic, clinical, and geographic factors associated with HPV detection to provide region-specific epidemiological insights that may inform cervical cancer prevention strategies in Malaysia.

## Methodology

### Study design and method

A retrospective cross-sectional study was conducted involving women recruited through community-based screening programmes across multiple divisions in Sarawak, Malaysia. Data were collected from Cytology Laboratory of Sarawak General Hospital (SGH). All women who participated in community-based screening programmes organised by government primary healthcare facilities (Klinik Kesihatan) across Sarawak between December 2024 and January 2026 were included in this study. Screening forms which were incomplete were excluded.

During the screening program, samples were collected at local clinics before being coordinated and submitted through the respective divisional health offices (Pejabat Kesihatan Bahagian). Cervicovaginal specimens were collected using one of the following methods: either cervical vaginal swab for HPV testing, liquid-based preparation, or conventional pap smear. The sample was collected either by self-collection or by a healthcare provider (assisted). All collected specimens were transported to the central cytology laboratory at SGH for processing and analysis. HrHPV detection and genotyping were performed using the Anyplex™ II HPV HR Detection assay (Seegene, South Korea), a multiplex real-time polymerase chain reaction (PCR) assay. The assay detected 14 high-risk HPV (hrHPV) genotypes, including HPV16, 18, 31, 33, 35, 39, 45, 51, 52, 56, 58, 59, 66, and 68. Samples yielding hrHPV results were routinely repeated according to laboratory procedures. Samples that remained invalid after repeat testing were reported as invalid, and a request for repeat specimen collection was issued through the respective healthcare facility. However, information regarding subsequent repeat sampling was not available within the study dataset. Therefore, participants with persistently invalid hrHPV results (*n* = 54) were excluded prior to analysis. The hrHPV detection was defined as the presence of one or more of these high-risk HPV genotypes. Genotype distribution was analysed among hrHPV-positive samples.

Between December 2025-February 2026, secondary demographic and clinical data were collected retrospectively from screening documentation. Variables collected included age, ethnicity, residential division, education level, household income, occupation, hormonal status, parity, sample type, and screening history. Ethnic groups with fewer than 15 participants were grouped under a single “Others” category to ensure stability of estimates in subgroup analysis. These comprised multiple minority ethnic groups across the study population. Postpartum status was defined as women within 42 days following delivery, in accordance with clinical practice guidelines [15]. Pap smear results were also recorded, where available.

The primary outcome was hrHPV prevalence, defined as the proportion of participants with detectable hrHPV among the total number of screened individuals. Secondary outcomes included genotype distribution among hrHPV-positive participants, prevalence of multiple infections, and factors associated with hrHPV detection.

### Statistical analysis

Descriptive statistics were used to summarise participant characteristics and hrHPV prevalence. Continuous variables were expressed as mean (standard deviation), while categorical variables were presented as frequencies and percentages. The hrHPV genotype distribution was expressed as percentages among hrHPV-positive samples. Associations between participant characteristics and hrHPV detection were evaluated using univariable analysis and multivariable logistic regression. Adjusted odds ratios (adjOR) with 95% confidence intervals were reported. A p-value of < 0.05 was considered statistically significant.

Crude hrHPV prevalence was calculated overall and by division. To account for differences in age composition across divisions, age-standardized hrHPV prevalence was calculated using the direct standardization method, with the Sarawak population age distribution as the standard population. Age-specific hrHPV prevalence for each age group was calculated as ri = di / ni, where di represents the number of hrHPV-positive participants and ni the number of screened participants in age group i. Standard population weights were calculated as wi = Ni / sum(Ni), where Ni is the Sarawak population in age group i. The age-standardized prevalence was then obtained as sum(ri × wi).

### Ethical considerations

Ethical approval for this study was obtained from the Medical Research and Ethics Committee (MREC), Ministry of Health Malaysia (NMRR ID-25-03455-GBL). As this study involved retrospective analysis of de-identified secondary data, the requirement for informed consent was waived.

## Results

### Participants characteristics

Data of 7,781 female participants were entered from the screening forms involving participants recruited across Sarawak. The baseline demographic and clinical characteristics are presented in Tables 1 and 2 respectively. The mean age of the participants was 43.6 ± 9.9 years. Approximately one-third of participants were from Kuching (28.6%), followed by Miri (14.1%) and Sibu (10.1%). Participants represented a diverse population comprising nearly 48 ethnic groups, with Iban (36.9%), Malay (17.9%), Chinese (12.0%), and Bidayuh (11.4%) being the most common. Most participants had secondary or higher education and a monthly household income of less than RM4,000. The majority were housewives (63.5%), and most samples were obtained via cervical vaginal swabs (94.3%). Most participants were undergoing first-time screening (90.5%) and were premenopausal (57.7%). The mean parity was 3.0 ± 1.7, with only a small minority (< 2%) reporting vaginal symptoms and 73.8% with normal cervical appearance on examination. Cervical examination findings were unavailable for 25.8% of participants, reflecting incomplete documentation within the screening dataset.

**Table 1:** Demographic characteristics of study participants (*n* = 7781)

Age, mean (SD)	43.55 (9.9)
Ethnicity, n (%)	
Malay	1395 (17.9)
Chinese	936 (12.0)
Indian	16 (0.2)
Iban	2872 (36.9)
Bidayuh	888 (11.4)
Kayan	130 (1.7)
Melanau	323 (4.2)
Penan	122 (1.6)
Kelabit	17 (0.2)
Kenyah	131 (1.7)
Lun Bawang	58 (0.7)
Berawan	15 (0.2)
Kedayan	43 (0.6)
Dusun	17 (0.2)
Bisaya	16 (0.2)
Others	117 (1.5)
Missing	685 (8.8)
Division, n (%)	
Betong	274 (3.5)
Bintulu	662 (8.5)
Kapit	341 (4.4)
Kuching	2225 (28.6)
Limbang	256 (3.3)
Miri	1101 (14.1)
Mukah	363 (4.7)
Samarahan	549 (7.1)
Sarikei	413 (5.3)
Serian	528 (6.8)
Sibu	786 (10.1)
Sri Aman	280 (3.6)
Missing	3 (0)
Education, n (%)	
No formal education	546 (7.0)
Primary school	1303 (16.7)
Secondary school	4152 (53.4)
Certificate	242 (3.1)
Diploma	577 (7.4)
Degree and above	635 (8.2)
Missing	326 (4.2)
Monthly household income, n (%)	
< 3999	6157 (79.1)
4000–7999	978 (12.6)
> 8000	226 (2.9)
Missing	420 (5.4)
Occupation, n (%)	
Government servant	885 (11.4)
Private sector	1056 (13.6)
Self-employed	426 (5.5)
Housewife	4940 (63.5)
Pensioner	83 (1.1)
Missing	391 (5.0)
Hormonal status, n (%)	
Pregnant	9 (0.1)
Postpartum	602 (7.7)
Pre-menopausal	4486 (57.7)
Menopausal	1819 (23.4)
No menses	3 (0.0)
Missing	862 (11.1)
Parity, mean (SD)	3.0 (1.7)

**Table 2:** Sample profile and symptoms of study participants

Sample type, *n* (%)	
Conventional pap smear	34 (0.4)
Liquid-based preparation	101 (1.3)
Cervical vagina swab	7340 (94.3)
Missing	234 (3.0)
Symptoms, n (%)	
Vaginal discharge	95 (1.2)
Contact bleeding	11 (0.1)
Laceration around cervix	5 (0.1)
Lower abdominal mass	1 (0.0)
PV bleeding	3 (0.0)
Abnormal bleeding	13 (0.2)
Cervix, n (%)	
Normal	5684 (73.0)
Abnormal	8 (0.1)
No cervix	82 (1.1)
Missing	25.8
Pap smear, n (%)	
NILM	467 (6.0)
ASC-US	26 (0.3)
LSIL	18 (0.2)
HSIL	8 (0.1)
Unsatisfactory	64 (0.8)
ASC-H	5 (0.1)
AGC-NOS	1 (0.0)
NA	7192 (92.4)
HrHPV Detection, n (%)	
Not detected	7008 (90.1)
Detected	718 (9.2)
Invalid	55 (0.7)
Multiple infection, n (%)	
Yes	115 (1.5)
No of infection, n (%)	
1	605 (7.8)
2	82 (1.1)
3	28 (0.4)
4	5 (0.1)

### HrHPV prevalence and genotype distribution

**Fig. 1:**
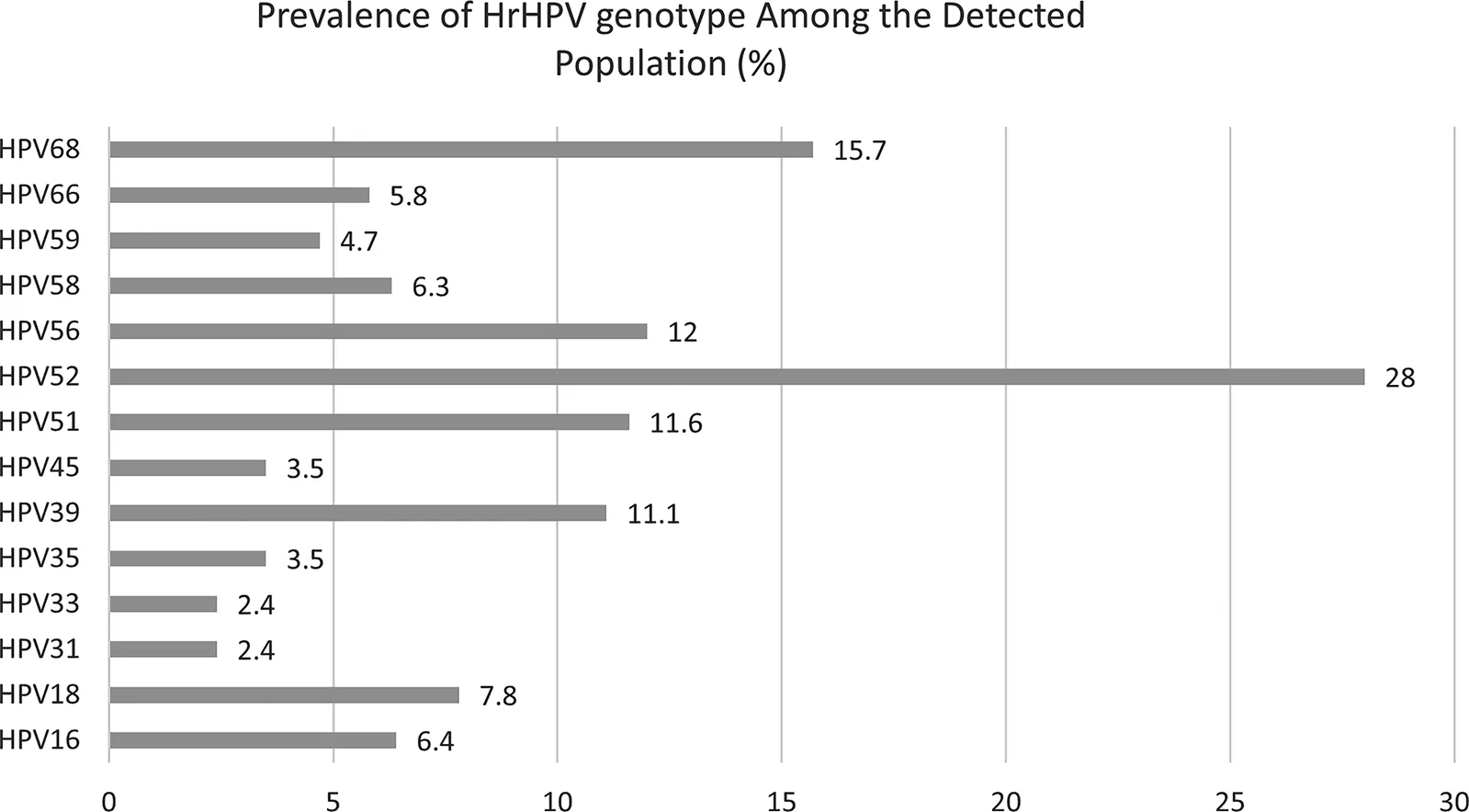
Prevalence of hrHPV genotype among the population who had HPV detected

Overall, hrHPV was detected in 718 participants, giving a prevalence of 9.2%, while 0.7% (*n* = 54) of results were invalid (Table 2). Among HPV-positive participants, 115 (1.5%) have multiple infections. The HPV subtype that had the highest prevalence among participants with positive HPV detection was HPV52 (28%), followed by HPV68 (15.7%) and HPV56 (12%) (Fig. 1). The highly oncogenic HPV16 and HPV18 accounted for 6.4% and 7.8% of detected infections, respectively.

### Papsmear results among HPV positive individuals

Pap smear data were largely incomplete, with 7,192 of 7,781 records (92.4%) unavailable (Table 2). Among HPV-positive participants, Pap smear results were retrievable for only 286 of 718 participants (39.8%). Therefore, the analysis of cytological findings among HPV-positive participants was based on a limited subset and should be considered exploratory. Among these 286 participants, 82.2% had negative for intraepithelial lesion or malignancy (NILM), while 17.8% had abnormal Pap smear findings. The abnormal findings comprised ASC-US (7.0%), LSIL (6.3%), HSIL (2.8%), and ASC-H (1.7%). These findings should be interpreted with caution, as the subset with available Pap smear results may not be representative of the overall HPV-positive population.

### Factors associated with HPV detection

In univariable analysis, age group, hormonal status, education level, and sample type were associated with hrHPV detection. After adjustment in the multivariable logistic regression model, only sample type remained significantly associated with hrHPV detection (Table 3). Missing data were handled using complete-case analysis; therefore, only participants with complete data for the outcome and all covariates included in the final model were analysed. Of the 7,781 participants in the full cohort, 6,536 were included in the final multivariable model, while 1,245 (16.0%) were excluded because of missing data. Complete-case analysis was used as a pragmatic primary approach. Although the final analytic sample remained substantial, exclusion of participants with incomplete data may have introduced bias if the missingness was not completely at random. To assess the robustness of the findings, supplementary reduced-model analyses were also performed after excluding selected covariates, including ethnicity and sample type, and the overall conclusions remained materially unchanged. Compared with the reference group, liquid-based preparation was associated with a higher likelihood of hrHPV detection. However, this finding should be interpreted cautiously, as this category contained relatively few observations, which may have affected estimate stability.

**Table 3:** Factors associated with HPV detection

	Not detected	Detected	*P* value	adjOR	*P* value
Age					0.582
< 45 yo	3984 (56.9)	455 (63.4)	0.003	1.2 (0.8-2.0)	0.366
45–59 yo	2491 (35.6)	220 (30.6)		1.1 (0.7–1.6)	0.613
>/=60yo (ref)	528 (7.5)	43 (6.0)			
Household incomes					0.930
< 4000 (ref)	5545 (83.6)	569 (83.2)	0.773		
4000–8000	879 (13.3)	96 (14.0)		1.0 (0.8–1.4)	0.721
> 8000	206 (3.1)	19 (2.8)		1.0 (0.6–1.7)	0.966
Hormonal status			0.042		0.674
Pregnant	8 (0.1)	1 (0.2)		1.6 (0.2–13.4)	0.661
Postpartum	544 (8.7)	56 (8.7)		1.1 (0.8–1.7)	0.577
Pre-menopausal	4007 (64.3)	445 (69.4)		1.2 (0.9–1.6)	0.242
Menopausal (ref)	1671 (26.8)	139 (21.7)			
Education					0.117
No formal or primary	1688 (25.1)	141 (20.5)	0.028	0.9 (0.6–1.2)	0.490
Secondary	3727 (55.5)	402 (58.5)		1.1 (0.9–1.5)	0.393
Tertiary (certificate, diploma, degree) (ref)	1305 (19.4)	144 (21.0)			
Sample type			< 0.001		< 0.010
Conventional pap smear	29 (0.4)	5 (0.7)		1.5 (0.5–4.4)	0.443
Liquid-based preparation	74 (1.1)	27 (3.9)		2.6 (1.4–5.1)	< 0.003*
Cervical vaginal swab for HPV (ref)	6622 (98.5)	665 (95.4)			
Sampling type			0.503		0.991
Self (ref)	5767 (87.9)	578 (87.0)			
Assisted by healthcare	791 (12.1)	86 (13.0)		1.0 (0.8–1.3)	
Ethnics			0.138		0.123
Malay (ref)	1272 (18.2)	119 (16.6)			
Chinese	834 (11.9)	99 (13.8)		1.4 (1.0–1.9)	0.028
Iban	2593 (37.0)	251 (35.0)		1.2 (0.9–1.5)	0.197
Bidayuh	788 (11.2)	98 (13.6)		1.4 (1.0–2.0)	0.027
Others	1521 (21.7)	151 (21.0)		1.1 (0.9–1.5)	0.364

* p-value < 0.05 indicates statistical significance

### Age-specific hrHPV prevalence

**Fig. 2:**
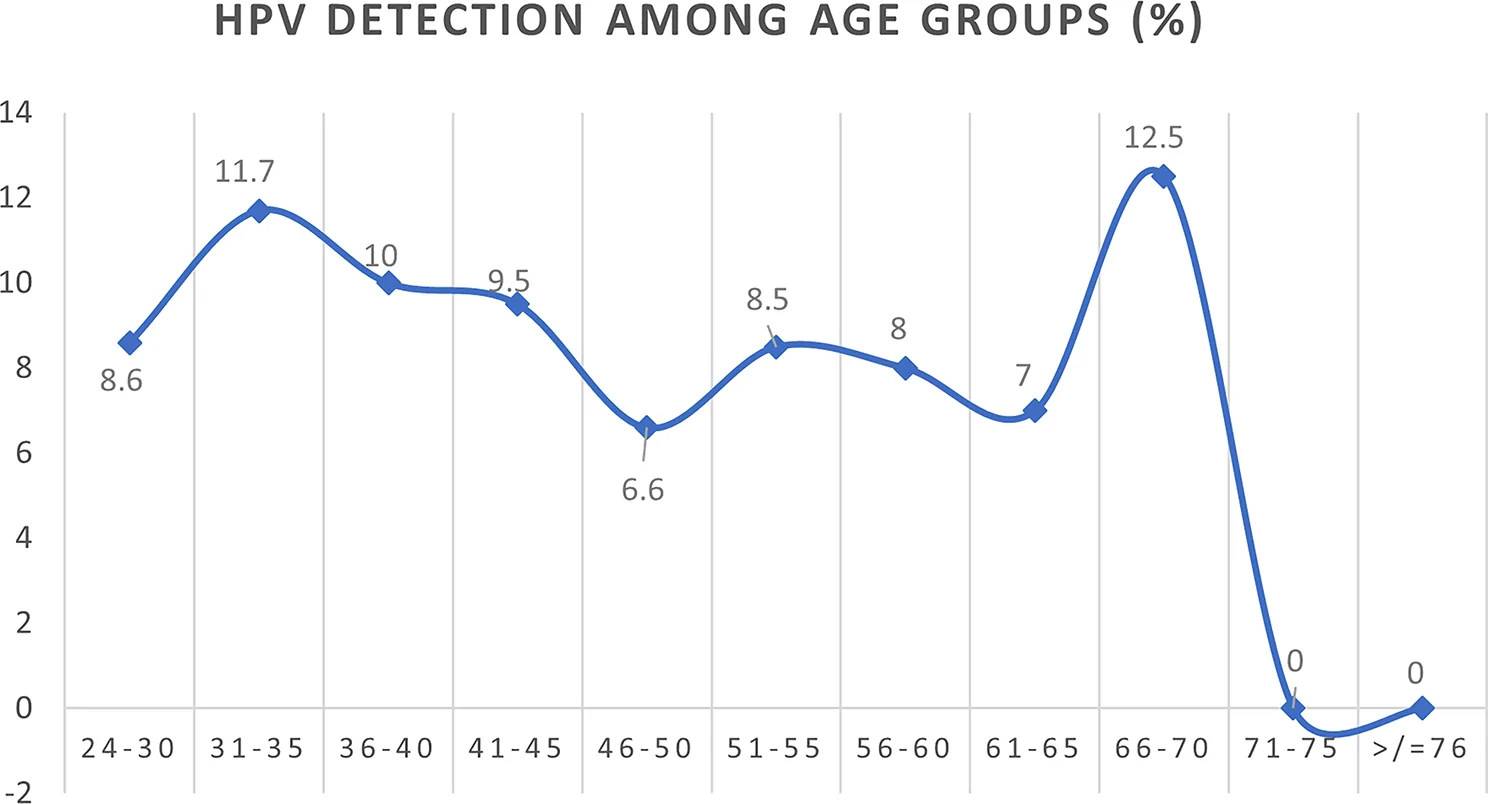
Age-specific prevalence of hrHPV infection among women participating in the Sarawak community-based screening programme

Age-specific hrHPV prevalence is shown in Fig. 2. HrHPV prevalence varied across age groups, with relatively high prevalence observed among women aged 31–35 years (11.7%). Thereafter, prevalence generally declined with increasing age, reaching 6.6% among women aged 46–50 years, before fluctuating modestly across older age groups. Although a prevalence of 12.5% was observed among women aged 66–70 years (2 out of 16), this finding should be interpreted with caution because of the relatively small number of participants in this age group. No hrHPV-positive participants were identified among women aged 71 years and above.

### Ethnic variation

**Fig. 3:**
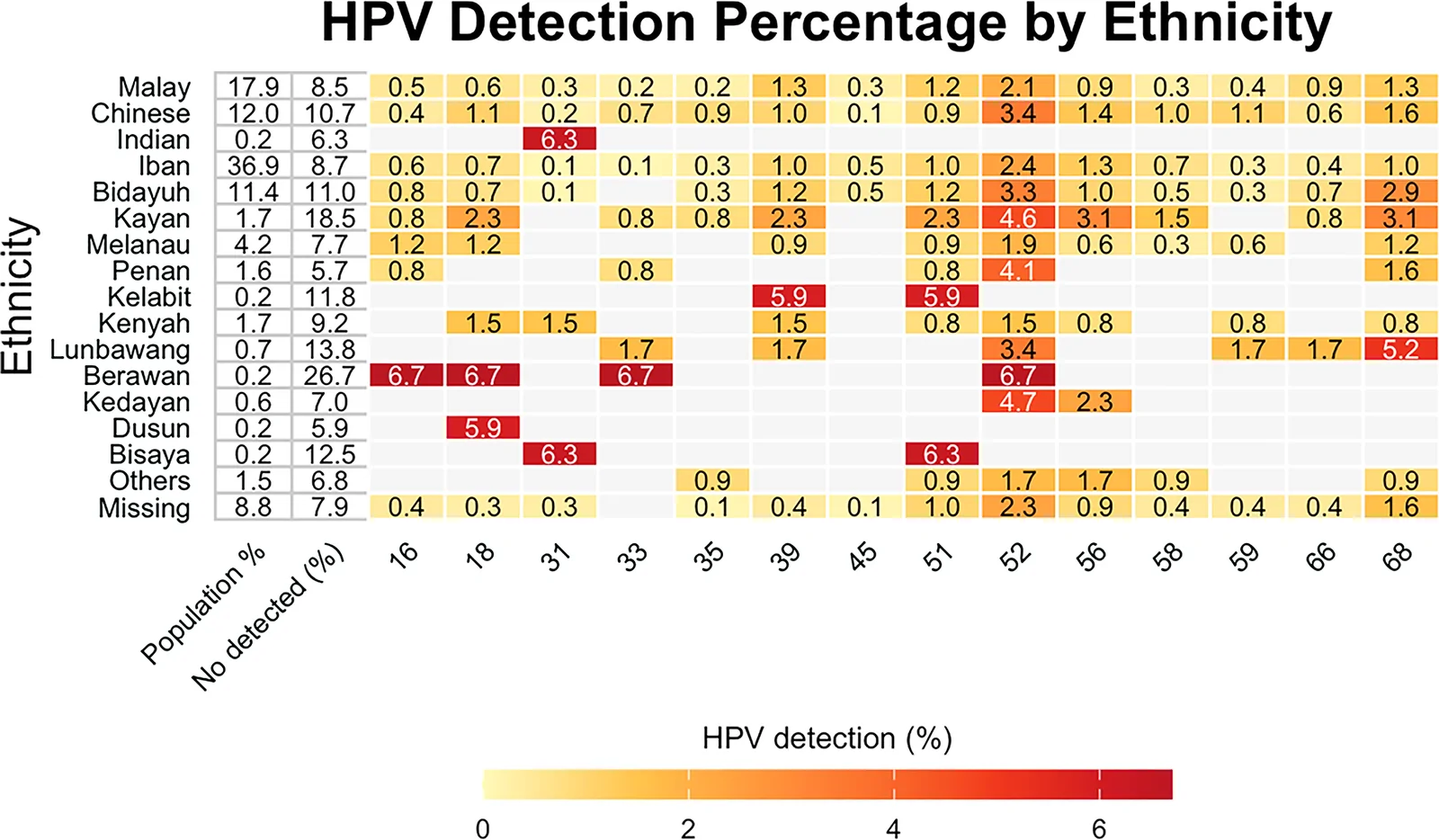
Heatmap showing the hrHPV Detection (%) by Ethnicity. There were near 48 different ethnic groups across the participants. Ethnic groups with fewer than 15 participants were grouped under “Others” to ensure stability of estimates. These included smaller ethnic populations such as Sunda, Selakau, Murut, Bugis, and other minority groups

Figure 3 presents a descriptive heatmap of hrHPV detection across ethnic groups and HPV genotypes. Ethnic groups with very small sample sizes (*n* < 15) were grouped under “Others” to improve estimate stability. Although higher detection percentages were observed in some smaller subgroups, including Berawan, Kayan, and Lun Bawang, these findings should be interpreted cautiously because of limited sample size. Accordingly, interpretation was focused primarily on the four major ethnic groups in the cohort, namely Iban, Malay, Chinese, and Bidayuh, each contributing at least 10% of the study population and therefore providing more stable estimates. Among these groups, hrHPV detection was highest in Bidayuh (11.0%), followed by Chinese (10.7%), Iban (8.7%), and Malay (8.5%). No formal statistical comparisons between ethnic groups were performed; therefore, these findings are descriptive and exploratory.

### Geographic variation

**Fig. 4:**
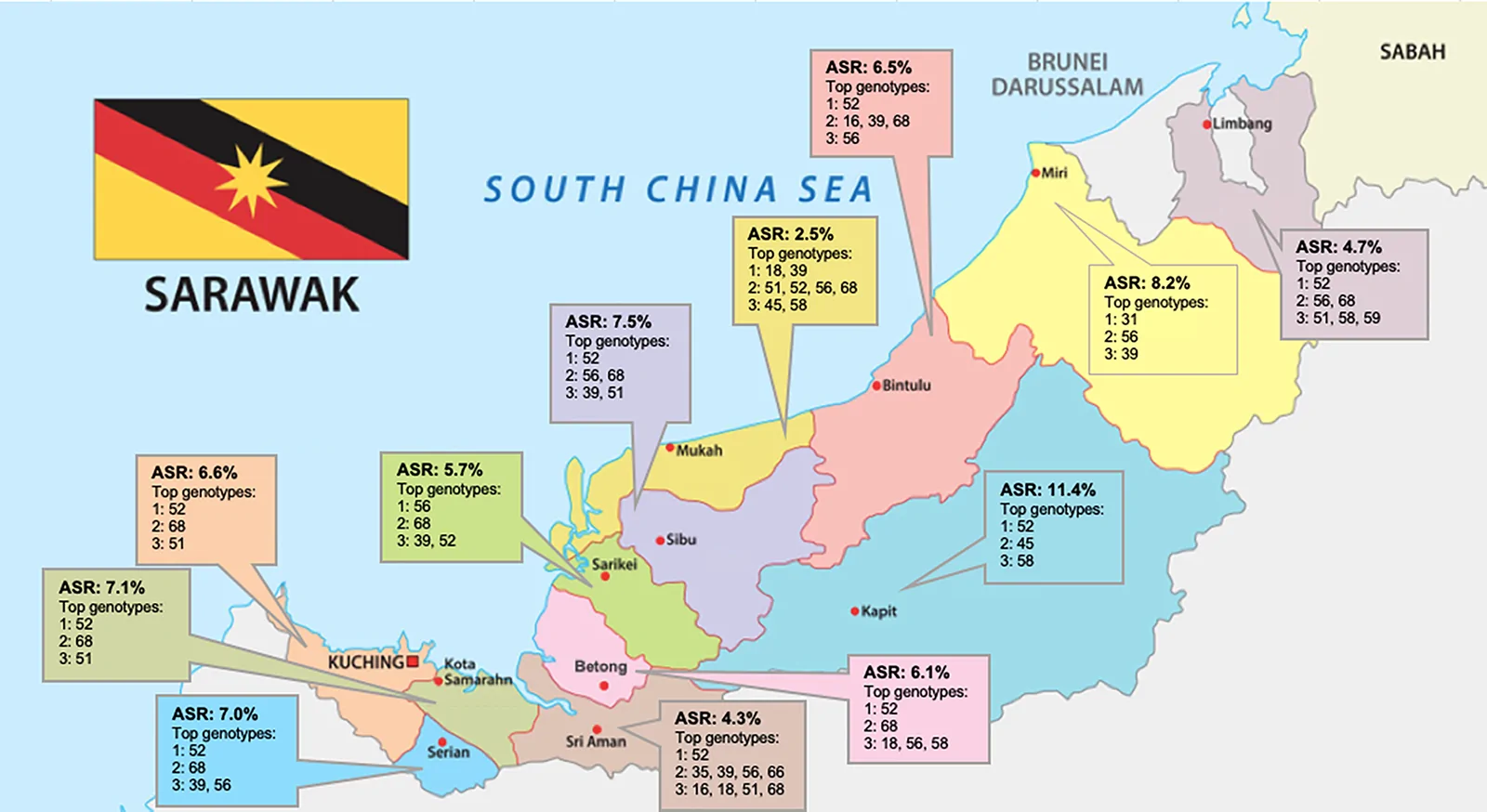
Age-standardized prevalence (ASR) of hrHPV infection across Sarawak divisions. The value shown for each division represents the ASR, while the accompanying labels indicate the three most frequently detected hrHPV genotypes

Geographic variation in hrHPV prevalence across Sarawak divisions is shown in Fig. 4. Crude hrhPV prevalence ranged from 3.6% to 11.7% across divisions. After age-standardization using the Sarawak female population, geographic variation remained evident, with ASRs ranging from 2.5% in Mukah to 11.4% in Kapit. Higher ASRs were also observed in Miri (8.2%), Sibu (7.5%), Samarahan (7.1%), and Serian (7.0%), whereas lower ASRs were noted in Sri Aman (4.3%) and Limbang (4.7%). In addition to variation in overall prevalence, differences in genotype distribution were observed across divisions. While HPV52 was consistently among the most frequently detected genotypes in multiple regions, other genotypes such as HPV68, HPV56, and HPV39 showed varying prominence across the state. Several divisions also demonstrated co-dominance of multiple genotypes, reflecting a heterogeneity in circulating hrHPV types throughout Sarawak.

## Discussion

The present study represented one of the largest community-based assessments of high-risk human papillomavirus (hrHPV) prevalence and genotype distribution in Sarawak, addressing a key gap identified in previous national-level analyses. Specifically, whereas prior investigations in Sarawak have largely been confined to small cohorts in isolated communities or hospital clinics, this study furnishes the largest community-based estimates to date, encompassing diverse ethnic groups and divisions across the state [2, 3, 16]. By leveraging data from a large screening cohort, this study provides important population-level insights into HPV epidemiology in an underrepresented region of Malaysia.

The overall hrHPV prevalence of 9.2% identified in this cohort is lower than that observed in other Asian populations, such as China, which reported a prevalence of 13.12%, but higher than the 7.2% prevalence documented in a multi-ethnic Malaysian population [17, 18]. Although the prevalence observed in this study is broadly consistent with the estimates reported in Malaysia, direct comparisons should be interpreted cautiously due to differences in study design, population characteristics, and HPV detection methods. Furthermore, direct comparisons between studies should be interpreted cautiously, as prevalence estimates may be influenced by differences in HPV detection assays, study populations, age ranges, sampling strategies, and screening settings. Notably, our findings extended previous national data by providing community-based estimates from Sarawak, a region that has been underrepresented in existing literature. This supported observations from our earlier systematic review, which highlighted a disproportionate concentration of HPV data from Peninsular Malaysia and limited representation from East Malaysia [3].

Age-specific analyses demonstrated that hrHPV prevalence was highest among women aged 31–35 (11.7%) and generally declined with increasing age, although a small apparent increase was observed among women aged 66–70 years. The latter finding should be interpreted cautiously due to small number of participants in this age group. Similar age-related patterns have been reported in large population-based studies in China, where HPV infection was more frequently detected among younger women and declined with increasing age, reflecting the natural history of HPV infection and immune clearance [19]. Furthermore, age-related differences in HPV genotype distribution have also been reported, suggesting that both HPV prevalence and genotype diversity may vary across different age groups [20]. These findings highlight the importance of continued HPV surveillance across all age groups to better understand the evolving epidemiology of HPV infection and to inform age-appropriate prevention strategies.

A key finding of this study is the predominance of HPV52, followed by HPV68 and HPV56, among hrHPV-positive participants. In contrast, HPV16 and HPV18, which are traditionally associated with cervical cancer globally, accounted for a smaller proportion of infections in this cohort. This genotype distribution differed from global patterns, where HPV16 and HPV18 remained the dominant types, but was consistent with reports from several Asian populations in which non-16/18 high-risk genotypes contribute substantially to HPV burden [5, 17]. For instance, HPV52, HPV58, and HPV16 were among the most common high-risk types identified in a prior Malaysian study [18], while another study in a suburban Sarawak population highlighted the endemic circulation of various genotypes [2]. These findings underscored the importance of region-specific HPV epidemiology. Furthermore, the high prevalence of non-16/18 hrHPV types, particularly HPV52 and HPV58, aligned with observations in other East Asian populations, suggesting a regional divergence from global HPV genotype distribution patterns [14, 21]. These findings highlighted the importance of region-specific HPV epidemiology and continued surveillance of circulating HPV genotypes.

Multiple hrHPV infections were identified in 16.0% of hrHPV-positive participants in this study, indicating that co-infection with more than one high-risk genotype is relatively common within the Sarawak population. The clinical significance of multiple HPV infections, however, remains incompletely understood. Some studies have reported that women with multiple hrHPV infections are more likely to present with abnormal cervical cytology, suggesting that co-infection may be associated with an increased risk of cervical abnormalities [22]. In contrast, other studies have observed that the prevalence of multiple HPV infections decreases with increasing severity of squamous epithelial abnormalities, suggesting that progression to high-grade lesions may be driven predominantly by the most oncogenic HPV genotype rather than the number of concurrent infections [23]. Collectively, these findings indicate that the role of multiple hrHPV infections in cervical carcinogenesis remains uncertain and warrants further longitudinal investigation to determine their prognostic significance and impact on cervical disease progression.

The observed genotype distribution has potential implications for HPV vaccination strategies. While current vaccines provide protection against HPV16 and HPV18, broader coverage vaccines include additional genotypes such as HPV52. This suggested that the implementation of a broader-coverage HPV vaccines may provide added benefit in populations where non-16/18 types contribute substantially to HPV burden [24]. The relatively high prevalence of HPV52 and other non-16/18 genotypes in this study suggested that vaccine strategies incorporating wider genotype coverage may be particularly relevant in this setting [25]. Furthermore, these findings reinforced the need for ongoing surveillance to monitor shifts in genotype distribution following vaccine implementation.

Liquid-based preparation was associated with higher likelihood of hrHPV detection compared with cervical vaginal swab in the present study. Previous studies have demonstrated that clinician-collected liquid-based cervical samples generally provide greater cellular adequacy and may yield improved analytical performance compared with self-collected vaginal samples, including lower cycle threshold values that reflect higher viral DNA concentrations [26]. Furthermore, liquid based cytology remains an effective method for cervical cancer screening, with studies reporting comparable clinical performance to primary HPV testing in selected screening populations [27]. However, this finding should be interpreted with caution, as only small proportion of participants in our study underwent liquid-based sampling. In addition, the observed association may partly reflect confounding by indication, whereby clinicians preferentially obtained liquid-based cervical samples from women perceived to be at higher clinical risk rather than representing an inherent advantage of the sampling method itself. Therefore, further studies with larger and more balanced sample sizes are required to better evaluate the influence of sample collection methods on hrHPV detection.

Although several demographic and clinical factors were evaluated, important behavioural and clinical risk factors for hrHPV infection, including sexual behaviour, smoking status, HIV infection, immunosuppression, and reproductive history, were not available in the study dataset. These factors are recognised determinants of HPV acquisition and persistence and may have influenced the observed associations, Consequently, residual confounding cannot be excluded. 

Recent studies from both Europe and Asia have demonstrated considerable geographic variation in HPV prevalence and genotype distribution [28, 29]. While HPV16 and HPV18 remain the predominant high-risk genotypes in many European populations (HPV16 accounting for up to 62.6% of cervical carcinomas), several Asian studies have reported a substantial contribution of non-16/18 genotypes, particularly HPV52 and HPV58, to the overall HPV burden [17, 28]. These regional differences underscore the importance of continued HPV surveillance, as shifts in genotype distribution may influence the long-term effectiveness of vaccination programmes and screening strategies. Consistent with these observations, geographic variation in hrHPV prevalence was observed across Sarawak divisions, with crude prevalence ranging from approximately 3.6% to 11.7%. After adjustment for differences in age distribution using age-standardized prevalence (ASR), geographic variation remained evident, with ASR values ranging from 2.5% in Mukah to 11.4% in Kapit. These findings suggest that the observed regional heterogeneity cannot be explained solely by differences in age composition across divisions. Other factors, including healthcare accessibility, screening uptake, sociodemographic characteristics, and behavioural factors, may also contribute to the variation in hrHPV prevalence across the state. Furthermore, differences in hrHPV genotypes distribution were observed across divisions, indicating regional heterogeneity in circulating hrHPV types throughout Sarawak. Similar regional disparities in HPV genotypes were also reported in China, where HPV52 and HPV58 frequently occur alongside HPV16 as dominant high-risk types [30, 31]. These findings emphasize the importance of regionally tailored vaccination and screening programmes that account for localized epidemiological patterns [32, 33]. Differences in hrHPV detection rates were observed across ethnic groups, however, these findings should be interpreted primarily as descriptive observations. Although higher detection rates were noted in certain ethnic groups, several of these populations were represented by relatively small samples sizes, limiting the precision of prevalence estimates. Furthermore, formal statistical comparisons between ethnic groups were not the primary objective of this study. Consequently, the observed differences should not be interpreted as evidence of statistically significant ethnic disparities in hrHPV prevalence. Similar variations in HPV prevalence and genotype distribution has been reported in other populations and may reflect a complex interplay of demographic, socioeconomic, behavioural and geographical factors rather than ethnicity alone [34, 35]. For example, variations in HPV prevalence and genotype distribution had been reported across different regions within China, attributed partly to the diverse ethnic populations and their distinct epidemiological characteristics [36]. Taken together, these findings highlighted the importance of population-specific surveillance to inform targeted and context-appropriate public health interventions [24, 37, 38]. The association between abnormal cytological findings and hrHPV detection observed in this study was consistent with established evidence linking persistent hrHPV infection to cervical epithelial abnormalities and cervical carcinogenesis [39, 40]. Consistent with previous studies, higher prevalence of hrHPV genotypes, including HPV16, HPV52, and HPV58, has been reported in samples with more severe cytological abnormalities, particularly in high-grade squamous intraepithelial lesions and cervical cancer [21, 41–43]. Similarly, HPV16 and HPV31 had been identified as primary causative agents of lesions in women presenting with abnormal cytology [44]. Collectively, these findings underscored the clinical relevance of HPV genotyping in cervical cancer risk stratification and screening strategies. Nevertheless, interpretation of the cytology findings in the present study should be made cautiously, as pap smear results were only available for a subset of participants and may not be representative of the overall study population.

## Conclusion

This study provided comprehensive community-based data on hrHPV prevalence and genotype distribution in Sarawak, addressing a key gap in HPV epidemiology in East Malaysia. HrHPV prevalence was moderate, with notable geographic and ethnic variation, although differences among major ethnic groups were modest. The predominance of non-16/18 genotypes, particularly HPV52, highlighted the importance of region-specific HPV surveillance and may have implications for vaccination and screening strategies.

### Limitations

This study has several limitations. First, the retrospective cross-sectional design precludes assessment of temporal relationships HPV persistence, progression to cervical disease, or differentiation between incident and persistent HPV infections. Second, incomplete pap smear data and missing cervical examination findings, largely due to incomplete documentation within the screening dataset, limited comprehensive evaluation of clinical outcomes and introduced the potential for selection bias in analyses involving cytology. Third, variation in sample collection methods may have influenced HPV detection rates, although this was partially addressed in the analysis. Fourth, information on important risk factors such as sexual behaviour, smoking status, HIV infection, immunosuppression, and reproductive history was not available in the dataset and therefore could not be included in the analysis. Consequently, residual confounding cannot be excluded. Fifth, participants were recruited through a community-based screening programme and may not be fully representative of the general population, potentially introducing selection bias. Sixth, geographic comparisons were based on crude prevalence estimates and were not adjusted for potential differences in age distribution, ethnicity, or other population characteristics across divisions. Finally, some subgroup analyses, particularly among smaller ethnic groups, were limited by small sample sizes, reducing the precision of prevalence estimates and limiting the ability to perform robust statistical comparisons between ethnic populations. The multivariable analysis was based on complete-case analysis, which may have introduced bias if missingness was not completely at random. However, materially similar overall conclusions were observed across three different regression models, supporting the robustness of the main findings.

Despite these limitations, this study provided valuable insights into hrHPV prevalence and genotype distribution in Sarawak. The large sample size, community-based design, and inclusion of diverse populations strengthen the generalisability of the findings. Further research should focus on longitudinal studies evaluating HPV persistence, clearance, and genotype-specific disease progression. Integration of HPV genotyping with cytological, histological, and other population-specific factors may provide additional insights into the natural history of HPV infection and improve risk stratification for cervical cancer prevention strategies within this population. Such studies would provide important evidence to support future cervical cancer prevention, screening, and vaccination strategies in Malaysia.

## Supplementary Information

Below is the link to the electronic supplementary material.

Supplementary Material 1

## Data Availability

The dataset generated and/or analyzed during the current study are available in the [HPV Pathology SGH] repository, [https://www.kaggle.com/datasets/melissalimsiawhan/hpv-pathology-sgh]
