# Supplementary material for: Prevalence and genotype distribution of high-risk HPV in Sarawak, East Malaysia
Source: BMC Infect Dis. 2026 Jul 17;26:1799. doi: 10.1186/s12879-026-14020-z (PMC13660091; doi:10.1186/s12879-026-14020-z)
Supplement: Supplementary file 1 [file 12879_2026_14020_MOESM1_ESM.docx]

**Supplementary**

Supplementary Table S1: List of ethnic groups included under “Others”

| Ethnic Group | Number of Participants (n) |
| --- | --- |
| Sunda | 6 |
| Selakau | 1 |
| Murut | 4 |
| Bugis | 11 |
| Sekapan | 3 |
| Bumiputera Sarawak | 15 |
| Bajau | 5 |
| Jawa | 12 |
| Tagal | 3 |
| Sebob | 2 |
| Punan | 13 |
| Sino | 3 |
| Saban | 3 |
| Indonesian | 1 |
| Filipino | 1 |
| Eurasian | 1 |
| Rungus | 2 |
| Kiput | 1 |
| Bumiputera Sabah | 1 |
| Kiput | 1 |
| Beketan | 6 |
| Seping | 2 |
| Kadazan | 7 |
| Suluk | 2 |
| Lahanan | 3 |
| Toraja | 1 |
| Sikh | 2 |
| Kadazan-Dusun | 1 |
| Punjabi | 1 |
| Irranun | 1 |
| Kejaman | 1 |
| Lun Dayeh | 1 |
